# SIBLING-TO-SIBLING DEMENTIA CARE: UNDERSTANDING MOTIVATIONS, CHALLENGES, AND EXPERIENCES

**DOI:** 10.1093/geroni/igae098.1318

**Published:** 2024-12-31

**Authors:** Jyoti Savla, Karen Roberto

**Affiliations:** Virginia Tech, Blacksburg, Virginia, United States; Virginia Tech, Blacksburg, Virginia, United States

## Abstract

Caregiving research has traditionally focused on older adult siblings’ involvement in shared social activities and emotional support, while offering limited exploration of instrumental help provided by siblings when their brothers/sisters face a health-related event or long-term illness. Moreover, there is a notable gap in understanding how siblings navigate the complexities of caring for their community-dwelling sibling living with dementia (SLwD). We begin to address this gap by examining determinants of sibling dementia caregiving roles, individual and family challenges older adult sibling caregivers encounter, and factors that facilitate their provision of care. From a mixed-methods analysis of interviews with 25 sibling caregivers, comprised of 12 sisters caring for sisters, 10 sisters caring for brothers, and 2 brothers caring for brothers (Mage = 68.03 years, R = 52-83; 48% African American; 52% co-residing), several key insights emerged. Motivations for assuming the caregiving role included shared life experiences, availability due to non-employment, personal or professional caregiving experience, and the absence or limited involvement of the SLwD immediate family. Early-life relationship conflicts, challenges in managing dementia-related symptoms, and divided family loyalties added complexity to the caregiving role. Sibling caregivers who received support from other family members and paid caregivers in caring for SLwD reported lower role captivity and caregiver burden. Caregiving experiences intersected with birth order, race, and socioeconomic status. Discussion focuses on when and why siblings take on the caregiving role for their SLwD, nuanced differences on how they manage their caregiving responsibilities, and implications for extended family caregiving practices and support.

